# Neural substrates of driving behaviour

**DOI:** 10.1016/j.neuroimage.2007.02.032

**Published:** 2007-05-15

**Authors:** Hugo J. Spiers, Eleanor A. Maguire

**Affiliations:** Wellcome Trust Centre for Neuroimaging, Institute of Neurology, University College London, 12 Queen Square, London WC1N 3BG, UK

**Keywords:** Virtual reality, fMRI, Verbal report, Action planning, Motor control, Rules, Monitoring, Traffic, Driver behaviour

## Abstract

Driving a vehicle is an indispensable daily behaviour for many people, yet we know little about how it is supported by the brain. Given that driving in the real world involves the engagement of many cognitive systems that rapidly change to meet varying environmental demands, identifying its neural basis presents substantial problems. By employing a unique combination of functional magnetic resonance imaging (fMRI), an accurate interactive virtual simulation of a bustling central London (UK) and a retrospective verbal report protocol, we surmounted these difficulties. We identified different events that characterise the driving process on a second by second basis and the brain regions that underlie them. Prepared actions such as starting, turning, reversing and stopping were associated with a common network comprised of premotor, parietal and cerebellar regions. Each prepared action also recruited additional brain areas. We also observed unexpected hazardous events such as swerving and avoiding collisions that were associated with activation of lateral occipital and parietal regions, insula, as well as a more posterior region in the medial premotor cortex than prepared actions. By contrast, planning future actions and monitoring fellow road users were associated with activity in superior parietal, lateral occipital cortices and the cerebellum. The anterior pre-SMA was also recruited during action planning. The right lateral prefrontal cortex was specifically engaged during the processing of road traffic rules. By systematically characterising the brain dynamics underlying naturalistic driving behaviour in a real city, our findings may have implications for how driving competence is considered in the context of neurological damage.

## Introduction

Driving a vehicle is an indispensable daily behaviour for many people. For some it represents their primary means of mobility, for others, their entire livelihood. Driving engages diverse cognitive functions such as perception, attention, motor control, working memory and decision making. How these complex operations are integrated during driving has been the focus of a number of psychological models (Groeger, 2000; Michon, 1989; Ranney, 1994). Michon (1989) has argued that driving processes can be grouped in three interacting hierarchical levels. The top level deals with strategic processes such as route choice or consideration of road traffic rules. The middle tactical level involves processes such as planning actions or adapting to the movements of other drivers. The lowest level is concerned with action execution and perceptual processing. However, despite the existence of such models and the importance of driving in daily life, little is known about how the brain supports driving behaviour. This is surprising given the lethal consequences of road traffic accidents and the importance of determining the impact of brain injury or dementia on driving capability (de Simone et al., 2006; Tamietto et al., 2006; Uc et al., 2004, 2005).

A small number of neuroimaging studies have used driving simulators to examine brain activity evoked during driving (Calhoun et al., 2002; Graydon et al., 2004; Horikawa et al., 2005; Uchiyama et al., 2003; Walter et al., 2001). These studies have revealed a network of brain regions more active during driving than rest periods which included the parieto-occipital cortices, cerebellum and cortical regions associated with perception and motor control. Activation in these regions was generally attributed to increased demands on vision, motor skills and visuomotor integration. In addition, activity in frontal, parietal, occipital and thalamic regions was found to correlate with average driving speed (Calhoun et al., 2002; Horikawa et al., 2005). Horikawa et al. (2005) also found that the number of crashes was negatively correlated with activity in the posterior cingulate, while others observed that the ability to maintain a safe driving distance was negatively correlated with activity in anterior cingulate (Uchiyama et al., 2003).

While these studies have begun to identify brain regions associated with driving, measures averaged over blocks typically of 30–60 s duration can tell us nothing about how brain activity changes during the different cognitive processes that characterise driving on much finer time scales. In an attempt to examine dynamic pattern of brain activity during driving periods, Calhoun et al. (2002) employed an independent components analysis, which used variation in the activity itself to determine the specific ‘hidden’ temporal signals in the data. Using this approach they found that different areas of the brain exhibited distinct patterns of dynamic modulation during driving. However, this approach is limited by the fact that the dynamic changes are difficult to relate either the second-by-second behaviour or the specific cognitive processes engaged.

Another limitation of all previous neuroimaging studies of driving is their use of racetrack-like environments. Such simulations severely limit the opportunity to examine some of the key events that that occur during driving in the real world. Typically, they lack options in route choice, there is no requirement to turn at junctions, or to take account of pedestrians and fellow road users, nor is there a need to consider traffic regulations that govern road safety. Thus, how brain activity changes with respect to important events during the experience of driving in real world settings remains unknown.

**Fig. 1:**
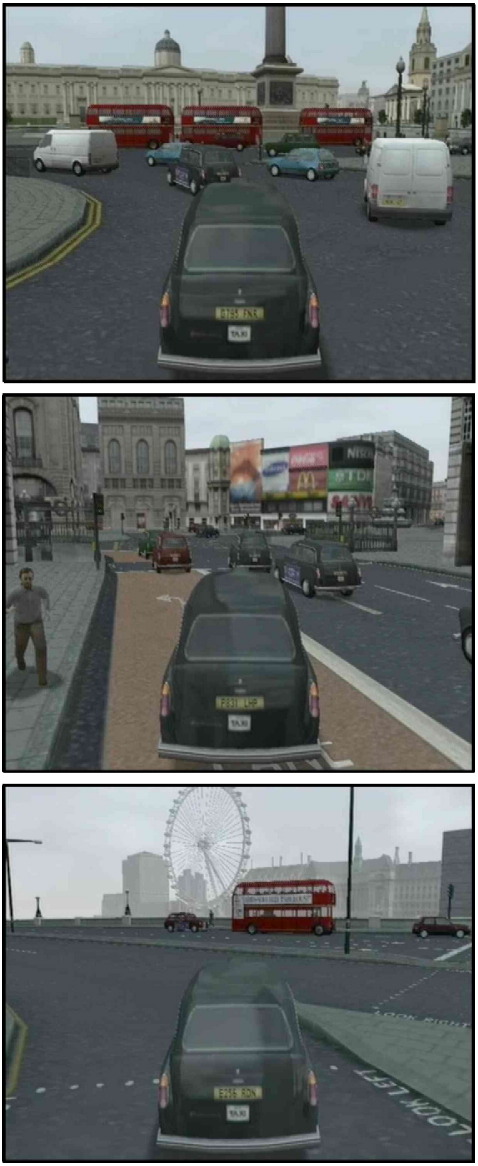
Example views from within the video game “The Getaway” © 2002 Sony Computer Entertainment Europe. Upper panel shows a view at Trafalgar Square, middle panel a view at Piccadilly Circus, lower panel a view looking towards the London Eye/Millennium Wheel. These images are reproduced with the kind permission of Sony Computer Entertainment Europe. The vehicle subjects drove during the experiment was a London taxi cab, visible in the lower middle of each image.

In this study, we over came the constraints of previous neuroimaging studies by adopting a novel approach. We examined brain activity associated with driving in a real world setting by scanning subjects with fMRI as they drove through an accurate simulation of a real city (London, UK, see Fig. 1). In the simulation, ordinance survey map data and video capture software were used to accurately recreate dimensions, textures and layout of over 110 km (70 miles) of drivable roads and numerous buildings. The one-way systems, working traffic lights, London’s traffic and an abundance of Londoners going about their business are all included, allowing us to examine driving in the very common context of a busy city. In order to investigate the brain activity associated with each subject’s actions at every second of their journeys, a video of a subject’s performance was recorded during scanning. By carefully examining the videos, we determined when subjects made particular actions and used this to model the fMRI data. We also employed a novel means of ‘reading’ participants’ thoughts while they moved around the city. To determine when subjects were engaged in different thought processes, immediately post-scan and without prior warning, subjects watched the video replay of their performance and were interviewed using a verbal report protocol that was carefully developed from pilot studies (Ericsson and Simon, 1980). Simply put, this involved getting subjects to review their performance and report on what they had been thinking while they had been doing the task in the scanner. The transcribed thoughts of the subjects could then be analysed and used with the record of their actions to model every second of the fMRI time series. By analysing the brain activity during specific driving actions and described thoughts, we were able to provide insight in the how the brain operates during the experience of driving through a familiar city.

## Materials and methods

### Participants

Twenty healthy right-handed male licensed London taxi drivers participated in the experiment (mean age 49.8 years, SD 8.5 years, range: 27–59 years). Licensed taxi drivers were chosen in order to ensure a consistent level of navigation experience in the environment. All subjects were naïve to the stimuli used in the experiment and gave informed written consent in accordance with the local research ethics committee. The subjects were all experienced drivers. Thus, this study does not address the issue of how motoring experience might impact on brain structure or function, although this is an interesting question for future research.

### The virtual environment

The video game ‘The Getaway’ (© Sony Computer Entertainment Europe 2002) run on a Sony Playstation2 (© Sony Computer Games Inc.) was used to present subjects with a ground-level first person perspective view of a simulation of central London, UK (see Fig. 1). The game designers decided to truly recreate the city and a large team of photographers walked the streets of central London for 2 years recording many streets, shops and other details. The simulated drivers and pedestrians followed the traffic regulations and reacted to the subjects’ movements appropriately, e.g., giving way at junctions. The ‘free roaming’ mode of the game was used, permitting free navigation with the normal game scenarios suspended. Subjects moved through the environment in a London taxi cab, controlled using a modified MRI-compatible game controller. The controller consisted of two joysticks. The right joystick provided analogue control of acceleration, braking and reversing and the left joystick control of steering. Joysticks were used rather than a steering wheel and pedals to reduce head-movement-related artefacts in the MRI signal. A ‘cheat’ modification was employed using Action Replay Max software (© Datel Design and Development Ltd. 2003), which ensured that contact between the subject’s vehicle and other vehicles did not result in a crash. Subjects were instructed to drive ‘legally’ as they would in actual London. All of the taxi drivers confirmed that the game was very reminiscent of their experience of driving in central London.

### Pre-scan training and familiarization

Two weeks prior to scanning, subjects were given 2 h of practice with the game controls by asking them to navigate to various locations in areas of environment not used in the experimental task. To avoid waiting for long periods at red traffic lights, subjects were familiarised with treating all traffic lights as green but were otherwise required to comply with all other road traffic regulations in the UK. Crashing into a wall in the environment caused the vehicle damage and the experiment to terminate, thus subjects were trained to avoid crashing. Thirty minutes before the scan, subjects were again given further practice in an area not used in experimental tasks. During this practice session, subjects were trained to respond to a set of recorded customers’ requests to take them to destinations in London. Finally inside the MRI scanner the subjects were given practice in an area of London not tested in the experimental tasks and with the MRI-compatible game control for between 2 and 3 min prior to the start of the experimental task. They were also given experience of hearing voices of customers over the noise of the scanner through head phones worn during the scan. Prior to scanning, subjects were told the locations they would be starting from in the experimental tasks but not the order.

### Experimental task

During fMRI scanning, driving was tested in blocks where subjects responded to customers’ requests (heard via head phones) by delivering them to their destinations. During each block one route was tested. When the game came on the screen, subjects were given between 3 and 5 s to orient themselves in the environment. Following this they heard a customer request a destination (mean duration 2.0 s). For all routes, at some point during the driving task subjects heard customers request a change of destination (mean duration 3.0 s) and on a separate occasion make a navigationally-irrelevant statement (mean duration 2.0 s). For three of the routes an additional request to avoid a location or go via a location was made by the customer (mean duration 3.7 s). Seven routes were tested. Two subjects completed only four routes, in one case due to discomfort, the other due to a technical problem. Each driving period ended when either the subject reached the destination or when a predetermined period of time elapsed. Each period of driving was separated by a period of rest in which the subjects viewed a blank white screen for 60 s. Total mean functional scanning time was 31 min 35 s (SD 4 min 9 s).

### Video recording

In order to debrief subjects and create an independent record of eye-tracking, two videos were recorded during the scan. Video output from the Playstation2 was split three ways: (1) to a projector presenting stimuli in the MRI scanner (view angle of 27.6°), (2) to a VHS video recorder for debriefing and (3) to a video mixer to create an eye-tracking video. Video output going into the video mixer was mixed with camera footage of the scan console and a stopwatch manually synchronised with the time stamp on debriefing video. By noting the time on the stopwatch when the scanner finished, it was possible to convert the time in the debriefing video into time from the onset of scanning. Gaze position cross hairs collected via an ASL504LRO infra red eye-tracker (Applied Science Laboratories, Bedford, MA) were overlaid onto the video sent to the mixed video recording. Accurately calibrated eye-gaze tracking was achieved in nine subjects.

### Verbal report protocol

Immediately post-scan the subjects were taken from the scan room to a separate room where they were given a surprise debriefing with a verbal report protocol (Ericsson and Simon, 1980). In this debriefing, subjects watched the video of their performance during scanning. They were carefully instructed to describe what they remembered thinking, step-by-step, during their original performance. The interview proceeded at a pace determined by the subject, with the video being paused and rewound by the interviewer where necessary to capture the details provided by the subject. A new copy of the original video was recorded during the interview with the voices of the subject and interviewer collected by a microphone overlaid. In accordance with the methods described by Ericsson and Simon (1980) and others (Jack and Roepstorff, 2003), the interviewer followed a predetermined protocol during the interview. The subject’s report was interrupted as little as possible, the interviewer intervening only to improve the subject’s specification of the onset and duration of thoughts where possible, and on occasion where clarification was required to latter aid analysis. The mean duration of the collection of the verbal reports was 108.9 min (SD 16.9 min).

### Analysis of the verbal reports and driving performance

Anonymised audio information from the verbal report interviews was transcribed by a professional transcription agency blind to the purpose of the experiment. By comparing a transcript with the time stamp from the original performance video, information about the timing of the thoughts was incorporated into the transcripts and any errors or unclear statements rectified. Each statement in the transcript was classified into one of a set of categories, and its onset and duration recorded to create a segmented timeline of the subject’s experiences in order to model the fMRI data. Unambiguous categories were predetermined by analysis of common repeated statements in the verbal reports of four subjects who took part in an in-depth pilot study outside of the scanner.

Driving-related thought categories included action planning, monitoring traffic and thoughts about the one-way system. Another category of thought common in the verbal reports was ‘coasting’, where subjects were actively driving through the city, but did not have any directed thoughts. This served as the baseline. Other categories of thoughts concerning spatial navigation and spontaneous mentalising were also identified and are reported elsewhere (Spiers and Maguire, 2006a,b). The independent eye-tracking video was used to aid the identification of onsets and durations where the subjects reported looking at moving vehicles, pedestrians and fixed features in the environment and served as an external measure to validate the procedure. In addition to the categories derived from the verbal report, it was possible to directly observe the subjects making actions during the driving task. These actions could be categorised as follows: starting to move, turning left, turning right, stopping, reversing, swerving, attempts to avoid collisions and actual collisions. The latter two categories were combined in the analysis due to the low number of events in each subcategory. The onset of the observed events was determined using the time stamp on the performance video.

### fMRI image acquisition and analysis

T2-weighted echo planar (EPI) images with BOLD (blood oxygen level dependent) contrast were acquired on a 1.5-T Siemens Sonata MRI scanner. We used standard scanning parameters to achieve whole brain coverage: 44 slices, 2 mm thickness (1 mm gap), TR 3.96 s, TE = 50 ms. The first 4 volumes from each session were discarded to allow for T1 equilibration effects. A T1-weighted structural MRI scan was acquired for each subject. Images were analysed in a standard manner using the statistical parametric mapping software SPM2 (www.fil.ion.ucl.ac.uk/SPM). Spatial preprocessing consisted of realignment, unwarping, normalisation to a standard EPI template in MNI space with a resampled voxel size of 3 × 3 × 3 mm and smoothing using a gaussian kernel with full width at half maximum of 10 mm. Following preprocessing, statistical analysis was performed using the general linear model. We used coasting as the baseline to which we compared each of the other driving-related events or epochs (see Table 1). Since we wished to compare categories represented as events and categories represented as epochs to coasting, two separate models were created. In one model coasting was modelled as epochs (mean duration 15.0 s, SD 2.6 s) and in the other as events. The coasting events were specified at the middle of each period of coasting rather than at the start or end to avoid temporal correlation of the other events/epochs in the other categories. The mean duration for monitoring traffic epochs was 6.4 s (SD = 1.7 s). Stopping was modelled as an event 3 s prior to the vehicle coming to a halt. This was based on the average time at which subjects were first observed breaking before stopping. In addition to the categories listed in Table 1, other spatial navigation categories (Spiers and Maguire, 2006a), theory of mind events (Spiers and Maguire, 2006b) and rest periods were also modelled as regressors of no interest. Categories consisting of events were modelled with a stick function, and categories consisting of epochs were modelled with a boxcar function (duration determined by the subject’s verbal report). In the case of each model, these functions were then convolved with the canonical HRF to create regressors. Importantly, all the categories were sufficiently uncorrelated in the time series to model them as separate regressors. Within each model, the subject-specific parameter estimates pertaining to each regressor (betas) were calculated for each voxel. The parameter estimates were entered into a second level random-effects analysis using *t*-tests. We report results in a priori regions of interest at *P* < 0.001 uncorrected for multiple comparisons, with an extent threshold of > 5 contiguous voxels. These regions were identified from previous neuroimaging studies of driving (Calhoun et al., 2002; Horikawa et al., 2005; Uchiyama et al., 2003; Walter et al., 2001) and form the basis of our discussion and interpretation of the data. For completeness in our results tables we also report other areas active at the same threshold of *P* < 0.001 uncorrected for multiple comparisons; however, these later activations should be interpreted with caution.

## Results

Aspects of the findings from this experiment relating to spatial navigation and theory of mind have been reported elsewhere (Spiers and Maguire, 2006a,b). We now report new analyses from this rich data set focused specifically on driving. In Spiers and Maguire (2006a), we briefly reported on the brain activity associated with action planning and monitoring traffic. Here we re-examine these concepts in depth within the context of driving, along with the other completely novel driving-related events.

### Behavioural and cognitive characteristics of driving in a city

Driving involves the dynamic interplay of a wide range of skills and cognitive processes, yet previous neuroimaging studies have tended to treat it as a single behavioural state (Calhoun et al., 2002; Graydon et al., 2004; Horikawa et al., 2005; Uchiyama et al., 2003; Walter et al., 2001). By using an accurate interactive simulation of driving in a bustling real city, a verbal report protocol and an in-depth analysis of behaviour, we now provide a detailed break down of the second by second actions and thoughts during driving (see Table 1). Initially, we must accelerate in order to start moving. Once in motion we must keep our vehicle headed in the correct direction. At many junctions we need turn into new streets, being careful to monitor our speed and rate of turn. Sometimes we must reverse, perhaps to park or make a three point turn. At the end of every drive, and at moments throughout, we need to bring the vehicle to safe stop. These actions – starting to move, turning corners, reversing and stopping – characterise the main repertoire of prepared actions during driving. However, not all actions during driving are prepared in advance. Occasionally, we must act to bring the vehicle under control when we swerve or try to avoid a collision. Quick reactions during these moments can make the difference between life and death. Furthermore, safe driving depends on more than executing sequences of actions, be they prepared or unprepared. Much of driving involves planning and monitoring, such as deciding when to change lanes at the appropriate time, observing the movements of other drivers in order to avoid future collisions, all the while taking into account road traffic regulations, such as one-way systems, in order to reach the destination safely.

### fMRI results

The fMRI time series were modelled in terms of the categories described in Table 1. The condition coasting, where subjects reported driving without any directed thoughts, was used as the baseline condition to which we compared the other categories in Table 1. Thus, we were able to discern what brain areas were engaged over and above those associated with movement through virtual London and operation of the games console that were also present during coasting. A full list of the locations of the peak coordinates from each the different contrasts can be found in Appendix A (Tables 2–12).

### Prepared actions

**Fig. 2:**
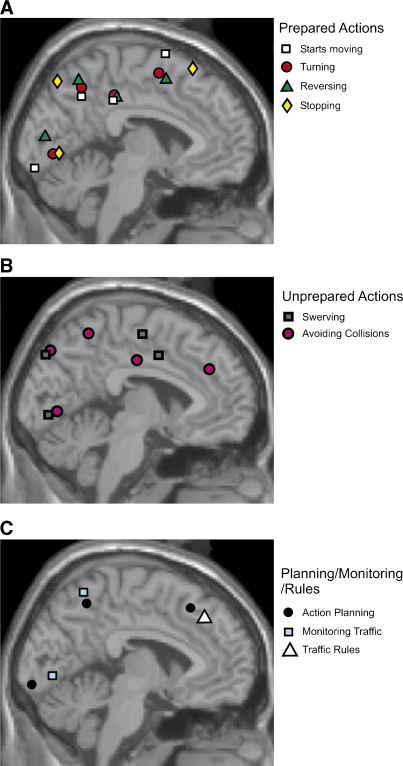
Locations of peak voxel activations lying on or near the midline for each condition versus coasting. See the Results section for details of activations in lateral regions and Appendix A for full details of peak coordinates and *Z*-scores. MNI coordinates are given here and all locations were identified at a threshold of *P* < 0.001 uncorrected. (A) Locations for peak coordinates (*x*, *y*, *z*) of activations during prepared actions are pre-SMA/SMA starts moving (SM) = 6, 9, 69, turning (T) = − 3, 0, 60, reversing (R) = − 3, 6, 54; stopping (ST) = 3, 12, 60; precuneus/parietal/posterior cingulate SM = 0, − 51, 45, T = 9, − 51, 54, R = 9, − 60, 60; ST = − 12, − 84, 48; posterior cingulate SM = − 6, − 24, 42, T = 9, − 27, 45, R = 12, − 27, 39; and medial occipital region SM = 6, − 81, − 9, T = 9, − 75, − 6, R = − 3, − 78, 15; ST = 15, − 69, 0. (B) Locations of peak coordinates for locations active during unprepared actions are SMA swerving (Sw) = 9, − 15, 51, anterior cingulate avoiding collisions (ACol) = − 3, 42, 33; mid cingulate Sw = − 6, 3, 42, ACol = − 9, − 21, 42; parietal cortex Sw = − 15, − 81, 39, ACol = 15, − 84, 36; and medial occipital cortex Sw = 0, − 75, 3, ACol = − 6, − 72, − 3. (C) Locations for peak coordinates of activations for the other categories of interest are pre-SMA action planning (AP) = 6, 21, 48, traffic rules = 6, 33, 42; medial parietal/precuneus AP = − 3, − 57, 51, monitoring traffic (MT) = 6, − 66, 51; medial occipital AP = 9, − 84, − 9, MT = 12, − 87, 3.

**Fig. 3:**
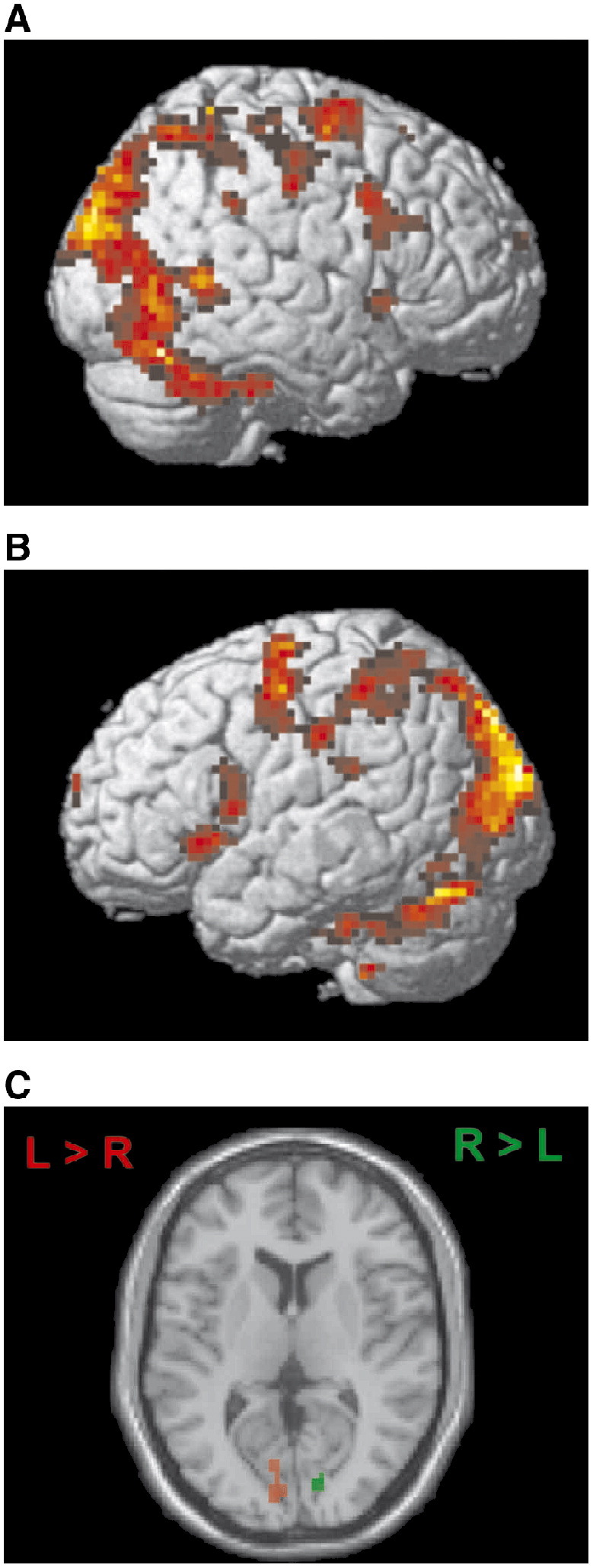
Brain activity related to turning corners. Significant activations for all turning events versus coasting events, shown for (A) the right hemisphere and (B) the left hemisphere. Images are shown at a threshold of *P* < 0.001. (C) Significant activation in left medial occipital cortex for turning left versus turning right and right medial occipital cortex for turning right versus turning left. Shown on the SPM2 template brain at a threshold of *P* < 0.05 corrected in order to illustrate the activity peaks more clearly.

Whenever subjects made any type of prepared action, increased activation was observed in a core network of brain regions including cerebellum, pre-supplementary motor area (pre-SMA)/SMA, posterior cingulate, medial parietal, medial and lateral occipital cortex (see Fig. 2A). Other brain regions were more active only during specific prepared actions. Starting moving was associated with significantly increased activation in much of the dorsolateral precentral gyrus and postcentral gyrus, with maximal response in the left dorsolateral precentral gyrus. Turning at junctions activated a widespread network, that also included activation of the dorsolateral precentral gyrus and extensive area extending from occipital cortex dorsally to superior parietal cortex and laterally in the right hemisphere to the posterior middle temporal gyrus (see Figs. 3A, B). Activation in the area of the calcarine sulcus ipsilateral to the direction of the turn was observed when turning in one direction was compared to turning in the opposite direction (see Fig. 3C). During periods of reversing activation was observed bilaterally in the lateral precentral gyrus and the anterior insula/ventrolateral prefrontal cortex (PFC). Stopping the vehicle was associated with activation in a more restricted network of regions than other prepared actions and the focus of activity in pre-SMA was found to be more anterior than other prepared actions (see Fig. 2A).

### Unprepared actions

**Fig. 4:**
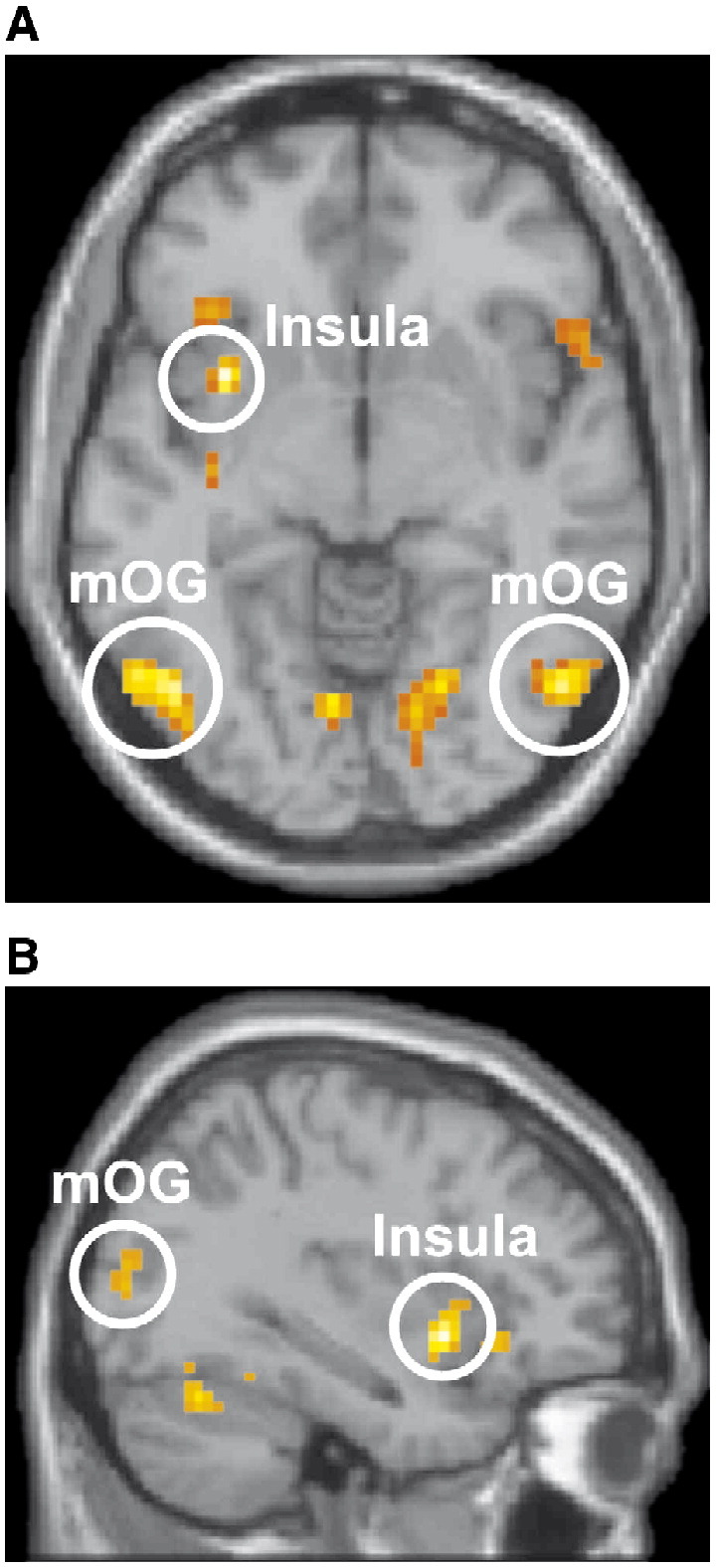
Brain regions more active during collision avoidance events than coasting events. Activations are shown on an axial (A) and sagittal section (B) of the SPM2 template brain at a threshold of *P* < 0.001. Regions circled are the middle occipital gyrus (mOG) bilaterally and the left anterior insula (insula). See Fig. 2B and Appendix A for details of other regions active.

Driving safely depends on making rapid responses. Whether controlling the vehicle in a swerve or acting to avoid a collision, activity in the cerebellum, medial occipital and posterior middle temporal gyrus was significantly increased during these moments (see Fig. 2B). Increased activity was also observed in the SMA and lateral dorsal premotor areas during swerving. Attempts to avoid collisions were additionally associated with activation in the mid and anterior cingulate, precuneus and posterior parietal cortex, bilateral ventrolateral PFC and left insula (see Figs. 2B and 4).

### Planning actions, monitoring actions and rule-related thoughts

**Fig. 5:**
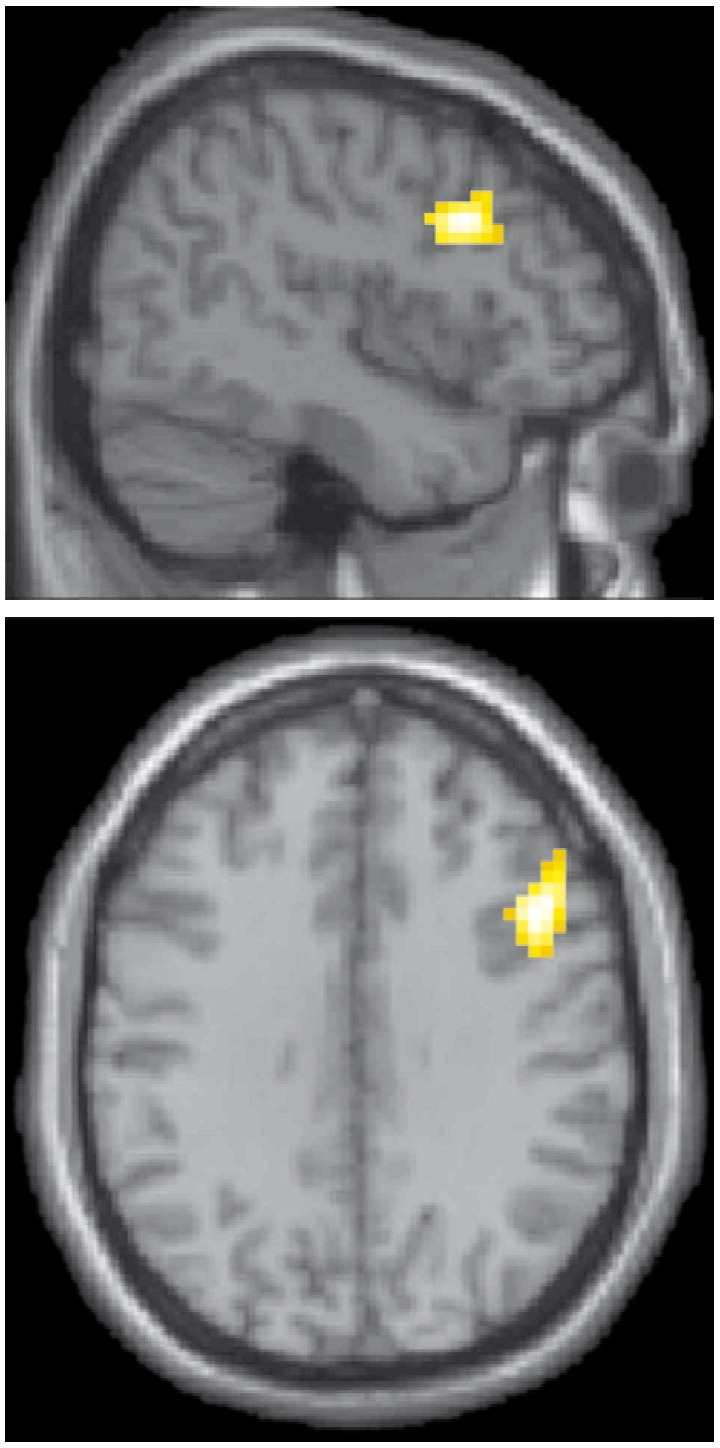
Activation in the right lateral prefrontal cortex during rule-related retrieval/processing relative to coasting events. Location of the peak coordinate in right lateral prefrontal cortex (45, 15, 33), shown at threshold of *P* < 0.001 on the SPM2 template brain. See also Fig. 2C for details of the activation in the pre-SMA.

Action planning was found to be associated with significantly increased activity in the pre-SMA, cerebellum, lateral occipital, superior parietal cortices and precuneus (see Fig. 2C). The activation in the area of the pre-SMA had a more anterior location to activations associated with either unprepared or prepared actions. Monitoring the actions of other drivers activated similar regions to action planning, but activation in the precuneus and superior parietal cortices was more extensive, and the activity in pre-SMA was not significantly increased (Fig. 2C). When subjects considered road traffic rules associated with the one-way system, significant activation was observed in the right lateral PFC (see Fig. 5) and at the most anterior limit of the pre-SMA (Fig. 2C).

## Discussion

In this study, we explored the second-by-second brain dynamics underlying the experience of driving in a simulated complex real city populated with people and moving traffic. We did this by combining an fMRI, an accurate interactive simulation of London (UK) and a retrospective verbal report protocol. This allowed us to probe the brain activity not only during the execution of specific actions, but also during moments of planning and monitoring actions, as well as when subjects were thinking about road traffic rules. Thus, our results extend those of previous neuroimaging studies which used racetrack-like environments and temporally gross measurements of performance to explore driving (Calhoun et al., 2002; Horikawa et al., 2005; Uchiyama et al., 2003; Walter et al., 2001). By examining specific events during driving our results provide insight into the brain regions supporting different components of driving described in Michon’s (1989) model of driving. Our categories, prepared actions and unprepared actions accord well with his operational level, action planning and monitoring traffic with his tactical level and thinking about road traffic rules with his strategic level.

### Performing prepared actions during driving

The processes of starting to move, turning corners, reversing and stopping are so familiar that we often fail to notice these complex acts during driving. The increased activity in pre-SMA/SMA, parietal and cerebellar regions common to all these actions is consistent with the suggestion that these regions play an important role in prepared movement execution (e.g., Cunnington et al., 2002; Deiber et al., 1991; Grafton et al., 1998; Ito, 2000; Nair et al., 2003). These regions have previously been found to be more active during blocks of driving than rest blocks (Calhoun et al., 2002; Horikawa et al., 2005; Uchiyama et al., 2003; Walter et al., 2001). Our findings now show that activity in these regions briefly increases specifically when these actions are performed. Observation of activation in pre-SMA is consistent with the suggestion that it is particularly important for performing prepared voluntary movements (e.g., Cunnington et al., 2002; Lau et al., 2004; Lee et al., 1999). The cerebellum is thought to be important for fine-control during movement execution (Horne and Butler, 1995), which would be vital when making any of the prepared maneuvers with the vehicle. Activity in medial and lateral occipital regions was also common to all the prepared actions. This likely relates to the known responsiveness of these regions to visual change (e.g., Tootell et al., 1998; Watson et al., 1993), which occur during such movements. In addition, increased demands on attention to visual motion and fixed landmarks during such actions may be associated with the increased occipital and parietal activity observed (e.g., Corbetta and Shulman, 2002).

While having some features in common, different prepared actions also have distinct characteristics, such as the way in which the driving controls must be coordinated or the type of visual motion. Thus, it is not surprising that, in addition to the common core network for prepared actions, increased activity in some brain regions was found to be specific to each of the prepared actions. The left lateral motor/premotor region was more active when starting to move, consistent with increased application of the accelerator (controlled by right hand) necessary to initiate movement of the vehicle. For practical reasons (see Materials and methods), a joystick was used rather than a foot pedal in our experiment. Had a foot pedal been used it is likely that activation of a more medial region of the primary motor cortex might have been observed. Activation in the calcarine sulcus in the ipsilateral hemisphere to the direction of the turn was observed when turning in one direction was compared with turning in the opposite direction. This may be due to differences in the direction of visual motion and accords with findings from a recent fMRI study which compared activity elicited by viewing visual motion in one direction with viewing it in the other direction (Bense et al., 2006).

Reversing was associated with increased activation of the lateral motor regions and the anterior insula. Activity in lateral motor regions may relate to the additional demands placed on motor control during reversing. Changes in the sensation of bodily orientation in the environment have been found to result in activation in the insula (Bottini et al., 2001) and hence in our study may be caused by the reversal of the direction of motion through the environment. Previous studies have shown that changes in medial premotor activity can be located more anteriorly the more it is prepared in advance (Lee et al., 1999). Thus, the more anterior focus of activation observed during stopping may relate to subjects possibly preparing to perform this action earlier than the other prepared actions.

### Responding to driving hazards

Driving can be a very dangerous, particularly when potential collisions must be avoided or when there is sudden loss of control. Determining the brain regions involved in reacting to these dangers may have particular implications for assessing the likely impact of brain damage on driving competence. Though the driving in our study was simulated, crashes had the potential to terminate the experiment, leading subjects to be highly motivated to avoid them. Our results show that activity in a number of brain regions was increased during events where subjects had to regain control during a swerve or had to avoid a collision. The quick reactions necessary during recovery from a swerve appear to be associated with increased activity in the SMA and lateral dorsal premotor regions. The more posterior distribution of the activity in SMA rather than pre-SMA accords with work showing that anterior–posterior distribution of activity in this region is sensitive to the level of preparedness of an action (Lee et al., 1999). The increased activity in posterior parietal cortex, medial occipital cortex and lateral posterior temporal gyrus when avoiding collisions may relate to the increased attention focused on a rapidly approaching object they wish to avoid. Activity arising in the mid cingulate and insula may be related to the particularly arousing nature of these events (Critchley, 2005). Horikawa et al. (2005) previously reported that activity in the right retrosplenial cortex was significantly correlated with the number of crashes made by subjects during simulated driving. We found no evidence that activity in this region increased during either avoiding collisions or swerving. Horikawa et al.’s (2005) findings were based on correlations across whole sessions and so likely had many influences in addition to crashes. We believe therefore that our event-related analysis yields more reliable evidence for the neural basis of crashes/near collisions. For practical reasons the visual experience of subjects was not immersive as it would be if they were actually driving in London. Thus, brain regions in addition to those reported here may play a role in detecting hazards appearing in the peripheral visual field.

### Planning and monitoring actions

The good driver plans actions in advance and keeps an eye on the movements of other drivers. Using a retrospective verbal report protocol, we were able to identify when subjects were engaged in action planning and monitoring traffic. Increased activity in the pre-SMA during action planning is consistent with the view that this region is important for selecting actions or the intention to make future actions (Lau et al., 2004; Lee et al., 1999). Increased activity in parietal cortex accords with the suggestion that it is important for future intentions (Andersen and Buneo, 2002) and planning actions (Milner and Goodale, 1995). The involvement of the lateral occipital cortex is interesting and not observed in many studies examining action preparation. This likely occurs because planned actions during driving typically involve focusing on locations on the road ahead thus giving rise to increased visual processing.

Monitoring traffic was associated with increased activity in the posterior parietal cortex, precuneus and lateral occipital cortex, consistent with previous reports of brain regions involved in visual and attentive tracking moving objects (Culham et al., 1998). The overlap between regions activated by action planning and monitoring traffic is interesting and shows that whenever subjects planned their own actions or observed the actions of others they recruited brain regions in a common network. However, unlike action planning, no increase in activity was observed in the pre-SMA/SMA, affirming the evidence that the pre-SMA/SMA has a greater role in our own actions rather than those of others.

### Road traffic rules

Safe driving in any city depends on abiding by the traffic rules, such as the use of one-way streets. All previous neuroimaging studies of driving by necessity had to neglect this aspect of driving due to their use of racetrack-like environments. In this study, subjects needed to consider the traffic regulations and this was found to result in increased activity in the right lateral PFC and medial PFC. This observation of lateral PFC is concordant with the suggestion that lateral PFC regions are important for rule retrieval and maintenance (Bunge et al., 2003; Donohue et al., 2005). Previous neuroimaging studies investigating rule use have tended to use abstract rules (Bunge et al., 2003) or test rule retrieval in an explicit manner (Donohue et al., 2005). In the present study, we were able to determine activity associated with the spontaneous processing of rules in the context of an interactive real world task. Activation of the medial PFC region on the boarder between the anterior cingulate and the pre-SMA may relate to the suggested role of this region in altering responses to adapt to challenges in the environment (Ridderinkhof et al., 2004). The observation of activity in prefrontal regions during our driving task argues against the suggestion from Walter et al. (2001) that the task of driving does not involve higher order brain regions.

### Conclusions

Driving is an almost ubiquitous behaviour. Researchers in many areas of cognitive neuroscience use analogies from driving to illustrate how their laboratory paradigms relate to the real world (see Groeger, 2000). Despite the broad interest in and the importance of driving to daily life, however, very little is known about how the brain supports driving behaviour. Our results now reveal the richness and dynamic nature of thought processes and actions associated with driving on a second by second basis and how activity in a number of brain regions changes in response to the many different challenges faced when driving through a busy city. Moreover, our findings may have implications for how driving competence is considered in the context of neurological damage, providing a framework for clinicians who are so often called upon to make judgements about a person’s fitness to drive.

## Figures and Tables

**Table:** 

Brain regions	*Z*-score	Coordinates of peak activation		
*x*	*y*	*z*
Pre-SMA/SMA	5.10	6	9	69
L precentral/postcentral gyrus	5.35	− 42	− 21	60
R precentral/postcentral gyrus	4.37	42	− 39	57
Medial occipital/calcarine sulcus	5.04	6	− 81	− 9
L cerebellum	4.90	− 30	− 66	− 33
R cerebellum	4.40	33	− 54	− 33
Precuneus/posterior cingulate	4.58	0	− 51	45
Posterior cingulate	4.50	− 6	− 24	42
R middle occipital gyrus	5.27	60	− 60	9
L superior temporal gyrus	4.25	− 63	− 42	12
R middle frontal gyrus	4.72	51	12	39
L middle frontal gyrus	5.13	− 48	24	27
R ventral insula	4.73	36	0	− 30
R ventromedial PFC	4.32	6	36	− 18
L head of caudate	4.04	− 3	3	− 6
R temporal pole	4.32	33	12	− 45
L temporal pole	3.85	− 51	9	− 15
L anterior thalamus	3.72	− 12	− 6	0

**Table:** 

Brain regions	*Z*-score	Coordinates of peak activation		
*x*	*y*	*z*
Pre-SMA/SMA	5.05	− 3	0	60
Medial occipital/calcarine sulcus	6.83	9	− 75	− 6
R precuneus/post. cingulate	6.35	9	− 51	54
L cerebellum	6.05	− 21	− 72	− 18
R cerebellum	5.10	27	− 66	− 21
L middle occipital gyrus	4.93	− 42	− 81	9
R middle occipital gyrus	4.80	48	− 69	− 3
L middle frontal gyrus	4.47	− 57	9	9
L anterior insula/ventrolateral PFC	4.35	− 39	21	− 3
R middle frontal gyrus	4.06	48	9	36
R insula	3.97	45	12	− 3
Frontopolar gyrus	3.71	0	66	18
L inferior parietal gyrus	3.52	− 60	− 30	27
R thalamus	3.41	15	− 15	6
R posterior cingulate	4.45	9	− 27	45
L posterior parietal cortex	6.01	− 24	− 81	36
R posterior parietal cortex	5.35	18	− 81	36
L precentral gyrus	4.48	− 39	− 9	57
R precentral gyrus	4.59	24	− 3	60

**Table:** 

Brain regions	*Z*-score	Coordinates of peak activation		
*x*	*y*	*z*
L medial occipital/calcarine sulcus	6.39	− 9	− 87	3
L postcentral gyrus	4.29	− 57	− 21	18
R middle occipital gyrus	4.13	33	− 87	18
L insula	4.93	− 36	− 9	− 9
R insula	3.52	39	− 12	− 3
R inferior temporal gyrus	3.79	60	− 6	− 33
R putamen	3.72	18	6	− 3
L putamen	3.46	− 24	0	− 3

**Table:** 

Brain regions	*Z*-score	Coordinates of peak activation		
*x*	*y*	*z*
R medial occipital/calcarine sulcus	5.34	12	− 84	6
R precentral gyrus	4.39	33	− 21	54
L superior frontal gyrus	3.49	− 15	− 9	57
R anterior orbital gyrus	3.63	21	48	− 9
R anterior middle frontal gyrus	3.47	42	42	24

**Table:** 

Brain regions	*Z*-score	Coordinates of peak activation		
*x*	*y*	*z*
Pre-SMA/SMA	4.13	− 3	6	54
L inferior PFC/anterior insula	5.11	− 54	9	12
R inferior PFC/anterior insula	4.50	51	21	− 3
L middle frontal gyrus	5.09	− 42	36	30
L supramarginal gyrus	5.00	− 60	− 36	24
L postcentral gyrus	4.33	− 42	− 36	54
R postcentral gyrus	4.83	66	− 24	30
R superior temporal gyrus	4.75	54	− 48	0
R posterior cingulate	4.80	12	− 27	39
R cerebellum	4.73	24	− 48	− 24
L cerebellum	3.88	− 24	− 75	− 24
Medial occipital cortex/cuneus	4.56	− 3	− 78	15
R caudate	4.21	18	6	12
Precuneus	4.20	9	− 60	60
R intraparietal sulcus	4.06	36	− 36	45
R superior precentral sulcus	3.96	48	0	45
R middle frontal gyrus	3.78	36	36	30
L anterior cingulate	3.75	3	45	6
L superior temporal gyrus	3.36	− 60	− 51	9

**Table:** 

Brain regions	*Z*-score	Coordinates of peak activation		
*x*	*y*	*z*
Pre-SMA	3.95	3	12	60
R medial occipital/lingual gyrus	5.32	15	− 69	0
R anterior insula	3.97	30	24	9
L cerebellum	3.91	− 36	− 66	− 33
R cerebellum	3.81	21	− 33	− 24
R middle frontal gyrus	3.86	21	6	60
L intraparietal sulcus	3.78	− 33	− 33	45
R inferior frontal gyrus	3.65	48	33	12
L superior parietal gyrus	3.54	− 12	− 84	48
R superior parietal gyrus	3.43	24	− 66	60
R posterior superior temporal gyrus	3.54	60	− 51	3
L inferior frontal gyrus	3.52	− 39	18	12

**Table:** 

Brain regions	*Z*-score	Coordinates of peak activation		
*x*	*y*	*z*
Medial occipital/calcarine sulcus	5.31	0	− 75	3
R middle occipital gyrus/pSTS	5.26	48	− 69	0
SMA/mid cingulate	4.25	9	− 15	51
R precentral gyrus	4.22	27	− 24	57
L precentral gyrus	3.55	− 24	− 12	57
R postcentral gyrus	4.25	63	− 15	24
L postcentral gyrus	4.19	− 60	− 24	21
L supramarginal gyrus	3.72	− 57	− 39	27
Mid cerebellum	4.15	0	− 66	− 33
L cerebellum	3.87	− 30	− 48	− 30
R cerebellum	3.80	30	− 42	− 30
L mid cingulate	3.81	− 6	3	42
L intraparietal sulcus	3.68	− 15	− 81	39
L middle occipital gyrus/pSTS	3.64	− 39	− 69	3
R inferior frontal gyrus	3.49	45	12	0
L brainstem	3.40	− 9	− 33	− 33

**Table:** 

Brain regions	*Z*-score	Coordinates of peak activation		
*x*	*y*	*z*
R posterior parietal cortex	5.03	15	− 84	36
L posterior parietal cortex	4.04	− 12	− 84	42
L anterior insula	4.84	− 33	9	− 6
L postcentral gyrus	4.61	− 15	− 48	66
R cerebellum	4.58	3	− 51	− 27
L medial occipital/calcarine sulcus	4.57	− 6	− 72	− 3
L middle occipital gyrus	4.55	− 42	− 78	3
R middle occipital gyrus	4.53	51	− 69	− 6
R fusiform	4.52	21	− 69	− 9
R superior parietal/precuneus	4.49	9	− 57	57
L supramarginal gyrus	4.46	− 57	− 33	42
R supramarginal gyrus	3.85	66	− 30	24
R inferior frontal gyrus	4.11	57	18	3
L mid cingulate	4.11	− 9	− 21	42
R mid cingulate	4.00	9	− 27	42
L posterior insula	3.96	− 36	− 15	− 3
L circular insula sulcus	3.90	− 39	0	− 24
L anterior cingulate	3.81	− 3	42	33
R middle frontal gyrus	3.79	45	6	36
L middle frontal gyrus	3.71	− 45	0	48
R intraparietal sulcus	3.71	33	− 33	39
L inferior frontal gyrus	3.59	− 36	24	− 6
L superior parietal gyrus	3.56	− 18	− 57	60
R superior frontal gyrus	3.45	6	− 15	72
L intraparietal sulcus	3.24	− 21	− 75	36

**Table:** 

Brain regions	*Z*-score	Coordinates of peak activation		
*x*	*y*	*z*
Pre-SMA	4.20	6	21	48
R cerebellum	5.13	39	− 51	− 30
L cerebellum	4.39	− 45	− 57	− 27
L superior parietal/precuneus	4.55	− 3	− 57	51
L inferior occipital gyrus	4.15	− 48	− 66	− 12
L intraparietal sulcus	4.12	− 18	− 78	33
R middle frontal gyrus (dorsolateral PFC)	4.05	51	9	42
R middle occipital gyrus	4.04	42	− 84	− 9
L middle occipital gyrus	4.04	− 45	− 75	12
L lingual gyrus	3.96	− 27	− 60	− 15
R medial occipital/calcarine sulcus	3.96	9	− 84	− 9
R superior frontal gyrus	3.76	18	6	66
R superior temporal sulcus	3.72	57	− 51	3
R middle temporal gyrus	3.68	48	− 66	− 9
R posterior parietal cortex	3.67	21	− 81	48
L superior frontal gyrus	3.62	− 21	9	60
L middle frontal gyrus (posterior)	3.62	− 36	− 6	54
L middle temporal gyrus	3.59	− 54	− 60	6

**Table:** 

Brain regions	*Z*-score	Coordinates of peak activation		
*x*	*y*	*z*
R superior parietal/precuneus	5.31	6	− 66	51
R inferior occipital gyrus	5.04	36	− 81	− 15
L inferior occipital gyrus	4.17	− 33	− 84	− 18
R middle frontal gyrus	4.54	48	15	27
R medial occipital/cuneus	4.50	12	− 87	3
L inferior lingual gyrus	4.38	− 21	− 60	− 12
L superior frontal gyrus	4.37	− 24	51	18
R superior frontal gyrus	3.92	27	54	9
R superior temporal gyrus	4.21	54	− 42	21
L medial occipital/calcarine sulcus	4.08	− 9	− 93	3
R cerebellum	3.94	39	− 45	− 39
L cerebellum	3.86	− 36	− 66	− 30
R intraparietal sulcus	3.85	42	− 42	45
L intraparietal sulcus	3.83	− 27	− 57	54
L superior parietal gyrus (posterior)	3.76	− 18	− 87	36
L lateral middle occipital gyrus	3.65	− 48	− 69	6
R lateral middle occipital gyrus	4.05	54	− 60	9
R posterior orbital gyrus	3.45	33	21	− 2
R superior occipital gyrus	3.55	21	− 96	21
L anterior insula/ventrolateral PFC	3.55	− 33	30	− 3
L thalamus	3.51	− 12	− 12	3
R superior precentral sulcus	3.42	33	− 3	54

**Table:** 

Brain regions	*Z*-score	Coordinates of peak activation		
*x*	*y*	*z*
R pre-SMA/medial PFC	4.55	6	33	42
R superior frontal gyrus	4.16	45	15	33

**Table 1:** Categories and their mean number of occurrences

Category	Mean number of occurrences (standard deviation)
Prepared actions	
Starts moving	15.0 (5.2)
Turning left	39.3 (6.2)
Turning right	37.8 (8.6)
Stopping	7.7 (4.8)
Reversing	5.3 (3.3)
Unprepared actions	
Swerving	12.0 (8.7)
Avoiding collisions	11.0 (4.4)
Planning/monitoring/rules	
Action planning	45.8 (15.1)
Monitoring traffic	11.4 (5.9)
Traffic rules	11.2 (5.7)
Baseline condition: coasting	25.8 (7.5)

## Appendix A Tables of peak coordinates and *Z*-scores

L = Left, R = right, PFC = prefrontal cortex, SMA = supplementary motor area, pSTS = posterior superior temporal sulcus. All activations are listed statistically significant at *P* < 0.001 uncorrected. For brevity, each region is only listed once: where several peaks were observed in the same region, the coordinates and *Z*-scores refer to the strongest activation. MNI coordinates are listed. Specification of the SMA and pre-SMA was guided by Picard and Strick (2001) and other regions by Duvernoy (1999).

Table 2 Starts moving > Coasting

Table 3 L and R turning > Coasting

Table 4 Turning L > Turning R

Table 5 Turning R > Turning L

Table 6 Reversing > Coasting

Table 7 Stopping > Coasting

Table 8 Swerving > Coasting

Table 9 Avoiding collisions > Coasting

Table 10 Action planning > Coasting

Table 11 Monitoring traffic > Coasting

Table 12 Traffic rules > Coasting
